# Barriers to Cervical Cancer Screening by Sexual Orientation Among Low-Income Women in North Carolina

**DOI:** 10.1007/s10508-024-02844-2

**Published:** 2024-04-16

**Authors:** Jennifer C. Spencer, Brittany M. Charlton, Peyton K. Pretsch, Phillip W. Schnarrs, Lisa P. Spees, Michael G. Hudgens, Lynn Barclay, Stephanie B. Wheeler, Noel T. Brewer, Jennifer S. Smith

**Affiliations:** 1 Department of Population Health, Dell Medical School, University of Texas at Austin, 1601 Trinity St., Bldg. A, Austin, TX 78712 USA; 2 Department of Population Medicine, Harvard Medical School and Harvard Pilgrim Health Care Institute, Boston, MA USA; 3 Department of Epidemiology, Gillings School of Global Public Health, University of North Carolina at Chapel Hill, Chapel Hill, NC USA; 4 Division of Pharmaceutical Outcomes and Policy, Eshelman School of Pharmacy, University of North Carolina, Chapel Hill, NC USA; 5 Lineberger Comprehensive Cancer Center, University of North Carolina at Chapel Hill, Chapel Hill, NC USA; 6 Department of Biostatistics, Gillings School of Global Public Health, University of North Carolina, Chapel Hill, NC USA; 7 American Sexual Health Association, Research Triangle Park, Durham, NC USA; 8 Department of Health Policy and Management, Gillings School of Global Public Health, University of North Carolina, Chapel Hill, NC USA; 9 Department of Health Behavior, Gillings School of Global Public Health, University of North Carolina, Chapel Hill, NC USA

**Keywords:** Cervical cancer, Cancer screening, Sexual orientation, Poverty, Human papillomavirus, Pap smear

## Abstract

We sought to examine cervical cancer screening barriers by sexual orientation among low-income women in North Carolina. The MyBodyMyTest-3 Trial recruited low-income women (< 250% of federal poverty level) aged 25–64 years who were 1+ year overdue for cervical cancer screening. We compared perceptions of cervical cancer screening among those who self-identified as lesbian, gay, bisexual, or queer (LGBQ; *n* = 70) to straight/heterosexual women (*n* = 683). For both LGBQ and straight respondents, the greatest barriers to screening were lack of health insurance (63% and 66%) and cost (49% and 50%). LGBQ respondents were more likely than straight respondents to report forgetting to screen (16% vs. 8%, *p* = .05), transportation barriers (10% vs. 2%, *p* = .001), and competing mental or physical health problems (39% vs. 27%, *p* = .10). Addressing access remains important for improving cervical cancer screening among those under-screened. For LGBQ women, additional attention may be needed for reminders, co-occurring health needs, and transportation barriers.

**Supplementary Information:**

The online version contains supplementary material available at 10.1007/s10508-024-02844-2.

## Introduction

For individuals in the USA who identify as lesbian, gay, bisexual, or queer (LGBQ) and have a cervix, the use of cervical cancer screening is low, with around 68% reporting being up to date on screening compared to 78% for screen-eligible straight persons with a cervix (McDonald et al., 2022; Suk et al., 2022). As cancer registries and large health record data do not routinely assess sexual orientation, the impact of lower screening use is poorly understood. The extant limited evidence suggests LGBQ individuals are at higher risk for cervical cancer than their straight peers (Herriges et al., 2022). Drivers of lower screening rates among LGBQ persons are not fully understood, but studies have suggested that differences in patient or provider risk perception due to fewer male-identified partners (Agénor et al., 2019), lack of provider recommendations (Solazzo et al., 2019), lower preventive and reproductive care use (Charlton et al., 2018; Tabaac et al., 2020), and stigmatizing healthcare experiences (Milner & McNally, 2020) may contribute.

Those living in poverty are also at high risk for cervical cancer (Boscoe et al., 2016; Spencer et al., 2021) and report lower screening rates due to financial and logistic barriers to care (Suk et al., 2022), but less is known about the intersection of barriers for those who are both low-income and identify as LGBQ. This is particularly important as we begin to focus on the so-called last mile, finding ways to improve screening access among the unscreened, a population typically experiencing multiple levels of marginalization (Rimel et al., 2022). This study aims to examine barriers to cervical cancer screening by sexual orientation in a cohort of low-income women.

## Method

### Participants

The MyBodyMyTest Phase 3 (MBMT-3) Trial recruited 759 low-income individuals with a cervix across 21 geographically diverse counties in North Carolina (a state in the southeastern United States) through clinic flyers, print and radio advertisements, and online ads seeking women who were overdue for a Pap smear and uninsured or on Medicaid (Spees et al., 2019). Eligible participants were aged 25–64 years, had household incomes below 250% of the federal poverty level (FPL), were not pregnant, and were at least a year overdue for cervical cancer screening; including at least 4 years since their last Pap smear or at least 6 years since their last HPV test by self-report (US Preventive Services Task Force et al., 2018). While study inclusion criteria and the participant survey did not address gender identity, recruitment materials referred to “women.” For this reason, we also use this term to refer to participants.

### Procedure

An initial screening by phone or in-person determined trial eligibility. After providing informed consent, participants completed a baseline phone survey and received a $25 incentive. Participants were then randomized to receive an HPV self-collection kit along with assistance scheduling a free clinic-based screening, or scheduling assistance only. The present analysis used data from the initial screening questions and baseline survey, both of which were completed before participants were randomized to a trial arm. Additional trial details are available in the full study protocol (Pretsch et al., 2023; Spees et al., 2019).

### Measures

Sexual identity was assessed through asking is participants identified as “heterosexual or straight,” “gay or lesbian,” “bisexual” or “other.” The one participant who selected “other” identified as “queer.” Six individuals declined to report their sexual orientation and were excluded from the analysis (analytic sample *n* = 753). We grouped together all LGBQ respondents and compared to heterosexual/straight respondents.

Participants reported race, ethnicity, education, income, and health insurance status in the initial phone screen. Additional descriptive characteristics assessed in the baseline survey included employment, marital or cohabitation status, smoking status, and self-rated general health and mental health. Birth control use included all short-acting hormonal contraception (oral contraception, injectable, and vaginal ring), long-acting hormonal contraception (hormonal implants and intrauterine devices), non-hormonal methods (sterilization, condoms, diagrams, withdrawal), and no method.

The baseline survey assessed perceptions of cervical cancer screening, access to care, and perceived barriers to cervical cancer screening. To examine barriers to screening, participants were asked the open-ended question, “What are some reasons that you haven’t had a Pap smear recently?” Participants could give multiple reasons, and each response was categorized by the team member conducting the phone survey into pre-specified categories, which included the following: cost, no insurance, no time/too busy, afraid/nervous, didn’t think about it, or forgot about it, no doctor, unsure of whether it was needed, or not feeling they needed a screening test. Additional reasons were recorded as “other” and were later recategorized into existing or new categories by consensus of the study team. We present only reasons endorsed by ten or more participants to reduce identifiability of participants.

The baseline survey assessed, using multiple choice questions, whether a doctor had recommended a Pap smear in the past year, whether women would likely get a Pap smear in the next 6 months, worry about getting cervical cancer, and how hard women felt it would be to get screening. Finally, the survey assessed the extent to which physical or mental health problems have kept them from getting cervical cancer screening. The full survey items appear in Supplemental Table 1.

### Analysis

Analyses compared demographic characteristics by sexual orientation using Fisher exact tests for categorical variables and independent group *t* tests for continuous variables. Analyses included “don’t know” or “refused” as an additional category. We report *p* values and describe “differences” qualitatively where *p* values are < .10. All statistical analyses were conducted using SAS software, version 9.4 (Cary, NC).

## Results

A total of 683 (90.7%) women identified as straight, and 70 (9.3%) identified as LGBQ (Table 1). The majority of LGBQ women identified as bisexual (*n* = 50), followed by lesbian or gay (*n* = 19), and queer (*n* = 1). LGBQ respondents were generally younger than straight respondents (median: 33 vs. 42 years, *p* < .001). Race and ethnicity varied by group, with LGBQ women more likely to report being non-Hispanic White (43.5% vs. 37.0%, *p* = .01). All respondents were low-income due the sampling and recruitment approach; therefore, household income and insurance coverage were similar by sexual orientation.

**Table 1:** Demographic characteristics of MyBodyMyTest-3 Trial participants by sexual orientation

	Straight, *n* = 683		LGBQ, *n* = 70	*p*
Sexual orientation				
Straight/heterosexual	683 (100%)		–	
Lesbian/gay	–		19 (27.1%)	
Bisexual	–		50 (71.4%)	
Queer	–		1 (1.4%)	
Median age in years (IQR)	42 (33–51)		33 (29–40)	< .001
Race and ethnicity				
Non-Hispanic White	253 (37.0%)		30 (43.5%)	.001
Non-Hispanic Black	327 (47.9%)		27 (39.1%)	
Hispanic or Latino	65 (9.5%)		2 (2.9%)	
Another race or ethnicity	38 (5.7%)		10 (14.5%)	
Missing	–		1	
Education				
Less than high school	71 (10.4%)		3 (4.3%)	.27
High school diploma or GED	230 (33.7%)		24 (34.3%)	
Some college, college degree, or higher	382 (55.9%)		43 (61.4%)	
Annual household income (thousands of US dollars)				
Mean, range	15k (0–75k)	12.9k (0–50k)		.45
Health insurance				.36
Insured (Medicare/Medicaid)	141 (20.7%)	19 (27.1%)		
Uninsured	540 (79.3%)	51 (72.9%)		
Missing	2	–		
Employment status				
Unemployed	380 (56.2%)	39 (56.5%)		1.00
Employed, part or full time	296 (43.9%)	30 (43.5%)		
Missing	7	1		
Marital status				
Single or never married	346 (51.2%)	52 (75.4%)		< .001
Married or cohabitating	141 (20.9%)	7 (10.1%)		
Divorced, separated, or widowed	189 (28%)	10 (14.5%)		
Missing	7	1		
Birth control used by self or partner				
None	414 (60.6%)	46 (65.7%)		.20
Short-acting hormonal contraceptives (pill, injectable, nuvaring)	18 (2.6%)	2 (2.9%)		
Long-acting hormonal contraceptives (IUD or norplant)	33 (4.8%)	7 (10.0%)		
Non-hormonal methods (sterilization, condoms, diaphragm, withdrawal)	213 (31.2%)	15 (21.4%)		
Any doctor/clinic/health care visits in the past year				
None	218 (31.9%)	22 (31.4%)		1.00
One or more	465 (68.1%)	48 (68.6%)		
Current smoking status				
Every day or some days	274 (40.5%)	40 (58.0%)		0.01
Not at all	402 (59.5%)	29 (42.0%)		
Missing	7	1		
General health (self-reported)				
Excellent or very good	238 (34.9%)	21 (30.0%)		.63
Good	200 (29.3%)	24 (34.3%)		
Fair or poor	244 (35.8%)	25 (35.7%)		
Missing	1	–		
Mental health (self-reported)				
Excellent or very good	365 (53.5%)	27 (38.6%)		.03
Good	138 (20.2%)	15 (21.4%)		
Fair or poor	179 (26.3%)	28 (40.0%)		
Missing	1	–		

“Another race or ethnicity” included participants who selected American Indian/Alaskan Native, Asian, Native Hawaiian/Pacific Islander, or multiple racial categories

*GED* General Education Development, *IUD* intrauterine device, *LGBQ* lesbian, gay, bisexual, or queer

Healthcare indicators, including having seen a doctor in the past year and method of birth control used, were similar by sexual orientation. LGBQ women were more likely than straight women to be current smokers (58.0% vs. 40.5%, *p* = .01). Self-reported general health was similar in both groups. Still, LGBQ respondents reported substantially worse mental health, with 40% reporting fair or poor mental health compared to 26.3% of straight respondents (*p* = .03).

**Fig. 1:**
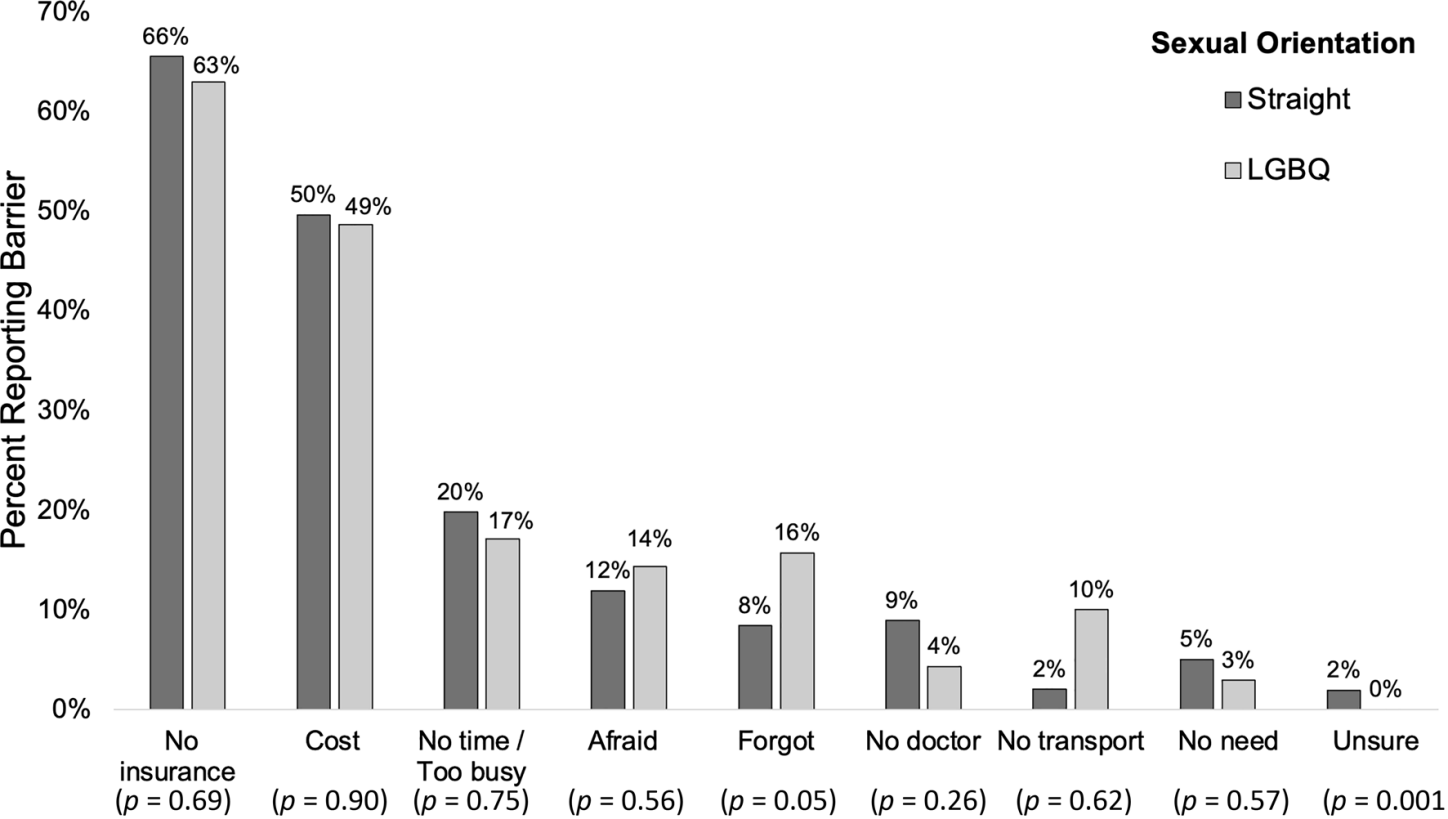
Barriers to cervical cancer screening by sexual orientation. Lesbian, gay, bisexual, or queer (LGBQ), *n* = 683; straight, *n* = 70

When asked “What are some reasons you have not had a Pap smear recently?”, both LGBQ and straight women most commonly reported insurance (63% LGBQ; 66% straight, *p* = .69) and cost (49% vs. 50%,* p* = .90) as barriers to cervical cancer screening (Fig. 1). Lack of time and fears related to testing were also commonly endorsed reasons. However, LGBQ respondents were twice as likely to say they forgot or did not think about testing compared to straight respondents (16% vs. 8%, *p* = .05) and much more likely than straight respondents to say transportation was a barrier (10% vs. 2%, *p* = .001).

Although all participants were overdue for screening, only a minority of both straight (24.0%) and LGBQ women (22.9%) heard from a provider in the past year that they should receive a Pap smear (Table 2) with 10% of LGBQ respondents versus 0.8% of straight respondents reporting that they didn’t know if one had been recommended (*p* < .001). Worry about cancer was similar in both groups, with 41.9% of straight and 45.7% of LGBQ women reporting that they were moderately or very worried about getting cervical cancer (*p* = .12).

**Table 2:** Perceptions of cervical cancer screening in the MyBodyMyTest-3 Study, stratified by sexual orientation

	Straight, *n* = 683	LGBQ, *n* = 70	*p*
Received Pap smear recommendation from doctor in last year			
Yes	164 (24.0%)	16 (22.9%)	< .001
No	513 (75.2%)	53 (67.1%)	
Don’t know	6 (0.8%)	7 (10%)	
Worry about getting cervical cancer			
Not at all or a little worried	395 (57.8%)	38 (54.3%)	.12
Moderately or very worried	286 (41.9%)	32 (45.7%)	
Refused/don’t know	2 (0.3%)	–	
Intentions to get a Pap smear in the next 6 months			
Probably will or definitely will	476 (69.7%)	51 (72.9%)	.68
Probably or definitely won’t	202 (29.6%)	19 (27.1%)	
Refused/don’t know	5 (0.7%)	–	
Difficulty of getting cervical cancer screening			
Not hard at all	286 (41.8%)	24 (32.4%)	.21
Somewhat, or very hard	374 (54.8%)	44 (64.7%)	
Refused/don’t know	23 (3.4%)	2 (2.9%)	
Physical or mental health problems interfered with getting cervical cancer screening			
Not at all	588 (72.3%)	43 (61.5%)	
A little, a moderate amount or a lot	184 (27.1%)	27 (38.5%)	.10
Refused/don’t know	4 (0.6%)	–	

*LGBQ* lesbian, gay, bisexual, or queer

The majority of both groups reported that they would probably or definitely get a Pap smear in the next 6 months (69.7 straight; 72.9% LGBQ; *p* = .68) but that they felt it would be somewhat or very hard to get screening (54.8% straight; 64.7% LGBQ; *p* = .21). LGBQ women were more likely to say that physical or mental health problems had kept them from screening (38.5%) than straight respondents (27.1%; *p* = .10).

## Discussion

We found important similarities in barriers for low-income women, comparing those who are LGBQ with those who are straight. For all respondents, lack of insurance and concerns about cost were the predominant reason for low screening rates. However, we found that low-income LGBQ women may face an additional set of barriers, as they were more likely than their straight counterparts to report that they did not think about screening, that mental or physical health got in the way of screening, or that they had transportation barriers.

Lower quality physical and mental health in LGBQ individuals has been reported by other studies (Charlton et al., 2018; Liu & Reczek, 2021; Potter & Patterson, 2019), and our findings support the idea that the higher prevalence of unmet health needs may produce additional barriers to seeking care or result in tradeoffs to prioritizing cancer screening, a particularly acute challenge for those without health insurance. Importantly, we did not find differences in the perceived risk of cervical cancer or intentions to screen by sexual orientation. Together, these findings suggest that interventions emphasizing enabling services, such as increasing accessibility of healthcare (Agénor et al., 2020), navigation services (Paskett et al., 2016; Roland et al., 2017), or addressing cultural competency of providers (Russell & Corbitt, 2022; Waryold & Kornahrens, 2020), may better address disparities than focusing on targeted educational efforts.

Previous studies have described barriers in primary care use (Macapagal et al., 2016) and reproductive healthcare for LGBQ individuals, including lower use of any hormonal birth control for lesbian and bisexual women relative to straight women (Agénor et al., 2021) and differences by sexual orientation in the choice of birth control methods (Charlton et al., 2018; Everett et al., 2018). We found the use of birth control was low but similar, for LGBQ and straight respondents, as was the use of healthcare in the past year*.* In both groups, around a quarter of respondents reported receiving a recommendation for a Pap smear in the past year, a notably low percentage given that all participants were overdue for screening, and more than two-thirds had a recent interaction with the healthcare system. Proactive outreach for sexual and reproductive care—including cervical cancer screening is important and should consider inclusive approaches to reach LGBQ populations (Jung et al., 2023).

Lastly, we note the high smoking rates in our sample. Smoking is a known risk factor for cervical cancer (Roura et al., 2014) and is more prevalent among both low-income and LGBQ individuals, with rates highest for those who are both (Blosnich et al., 2013). Improving available resources for smoking cessation and considering policy solutions to reduce smoking could improve overall health while also working toward cancer health equity.

Our population was a geographically diverse sample within the state of North Carolina, which, at the time of the study, was among the twelve US states that had not chosen to expand their Medicaid program, leaving many low-income individuals at higher risk of being uninsured and forgoing care due to cost (Spencer et al., 2019). Many of these states, North Carolina included, have few policies to protect the rights of LGBQ individuals, making the intersection of income and LGBQ identities particularly important in these settings (Agénor et al., 2022). As of 2023, North Carolina has passed, but not yet implemented, a plan for Medicaid expansion, offering a crucial opportunity to expand preventive care access to vulnerable populations across the state.

Our study should be considered in light of several limitations. First, prior studies have shown differences between minoritized sexual orientation groups (e.g., bisexual vs. lesbian) that we could not examine due to a smaller sample size (Solazzo et al., 2019, 2020). Similarly, the MBMT-3 Study did not assess gender identity, nor did it use gender-inclusive language in recruitment materials, which some transgender people may have seen as unwelcoming or not relevant to them. Transgender and nonbinary individuals with a cervix face additional cervical cancer screening barriers (Connolly et al., 2020) that are likely exacerbated for those living in poverty*.* To better understand these barriers, it is vital for more studies to collect sexual orientation and gender identity data, regardless of whether this is the study’s primary aim (Schabath et al., 2017). Beyond simply collecting data, incorporating an equity lens from the project’s onset would also strengthen future work. For example, researchers should use gender-inclusive language and assess experiences of homophobia and transphobia. To this point, important mediators such as provider trust and stigma in healthcare settings were not assessed in our study, so we cannot determine how much they explain cancer screening delays for low-income LGBQ individuals. However, we note that these were not mentioned by any participants in open-ended responses. Future work should consider incorporating validated measures of provider and healthcare system trust, discrimination, and stigma. Despite these limitations, our study provides an important and unique insight into barriers experienced by an understudied and historically marginalized population: low-income LGBQ individuals.

We identified lack of insurance and cost concerns as the main reasons for delaying screening among both straight and LGBQ individuals living in poverty. LGBQ individuals in our study also faced barriers to prioritizing preventive care related to co-occurring mental and physical health needs as well as forgetting to screen and experiencing transportation barriers. Interventions to increase cervical cancer screening in this population should center on cost and accessibility. To achieve equity in health outcomes, interventions should also consider additional enabling services to help overcome transportation barriers and ensure that materials, reminders, and study personnel focus on inclusivity in outreach and communication.

### Supplementary Information

Below is the link to the electronic supplementary material.

Supplementary file1 (DOCX 17 KB)

## Declarations

### Disclaimer

The NIH had no role in the study design, collection, management, analysis, interpretation of the data, writing of the manuscript, or decision to submit this report for publication.
