# Supplementary material for: Barriers to Cervical Cancer Screening by Sexual Orientation Among Low-Income Women in North Carolina
Source: Arch Sex Behav. 2024 Apr 16;53(5):1645–52. doi: 10.1007/s10508-024-02844-2 (PMC11106100; doi:10.1007/s10508-024-02844-2)
Supplement: Supplementary file 1 — Supplementary file1 (DOCX 17 KB) [file 10508_2024_2844_MOESM1_ESM.docx]

**Appendix Table 1: Relevant survey questions from the My Body, My Test study**

| **Question** | **Response Options** | **Analytic Approach** |
| --- | --- | --- |
| **What are some reasons that you haven't had a Pap smear recently?** | Open Ended | Responses categorized by interviewer as: Cost, no insurance, no time or too busy, afraid or nervous about the Pap (includes fear of discomfort, pain, embarrassment), afraid or nervous about the results or hearing she has cancer, didn't think about it or forgot about it, no doctor (never gets preventive health care or doesn't go to the doctor generally), unsure (lack of knowledge - didn't know needed, when needed, unsure of recommendation), doesn't need one (knows the recommendation but disagrees), refused, don't know, and other. Those categorized as "other" were further categorized by research team consensus; with transportation barriers as the most commonly reported “other” reason.  Reported each category with >10 responses. |
| **In the past year, has a doctor said you should get a Pap smear?** | Yes | Yes |
|  | No | No |
|  | *Refused* | Refused/Don't know |
|  | *Don't know* |  |
| **How worried are you about getting cervical cancer? Would you say you are…** | Not at all worried | Not at all worried - A little worried |
|  | A little worried |  |
|  | Moderately worried | Moderately worried - very worried |
|  | Very worried |  |
|  | *Refused* | Refused/Don't know |
|  | *Don't know* |  |
| **How likely are you to get a Pap smear in the next 6 months? Would you say you…** | Definitely won't | Definitely won't - Probably will |
|  | Probably won't |  |
|  | Probably will | Probably will - Definitely will |
|  | Definitely will |  |
|  | *Refused* | Refused/Don't know |
|  | *Don't know* |  |
| **How hard do you think it would be to get cervical cancer screening? Would it be…** | Not hard at all | Not hard at all |
|  | Somewhat hard | Somewhat hard- Very hard |
|  | Very hard |  |
|  | *Refused* | Refused/Don't know |
|  | *Don't know* |  |
| **How much have physical health problems or mental health problems kept you from getting cervical cancer screening?** | Not at all | Not at all |
|  | A little | A little - A moderate amount - A lot |
|  | A moderate amount |  |
|  | A lot |  |
|  | *Refused* | Refused/Don't know |
|  | *Don't know* |  |

*“Refused” and “Don’t know” were not provided to participants as response choices but were provided to interviewers to mark as appropriate.*
